# Supplementary material for: A systematic dissection of human primary osteoblasts in vivo at single-cell resolution
Source: Aging (Albany NY). 2021 Aug 24;13(16):20629–50. doi: 10.18632/aging.203452 (PMC8436943; doi:10.18632/aging.203452)
Supplement: Supplementary Figure 1 [file aging-13-203452-s001.pdf]

SUPPLEMENTARY FIGURE

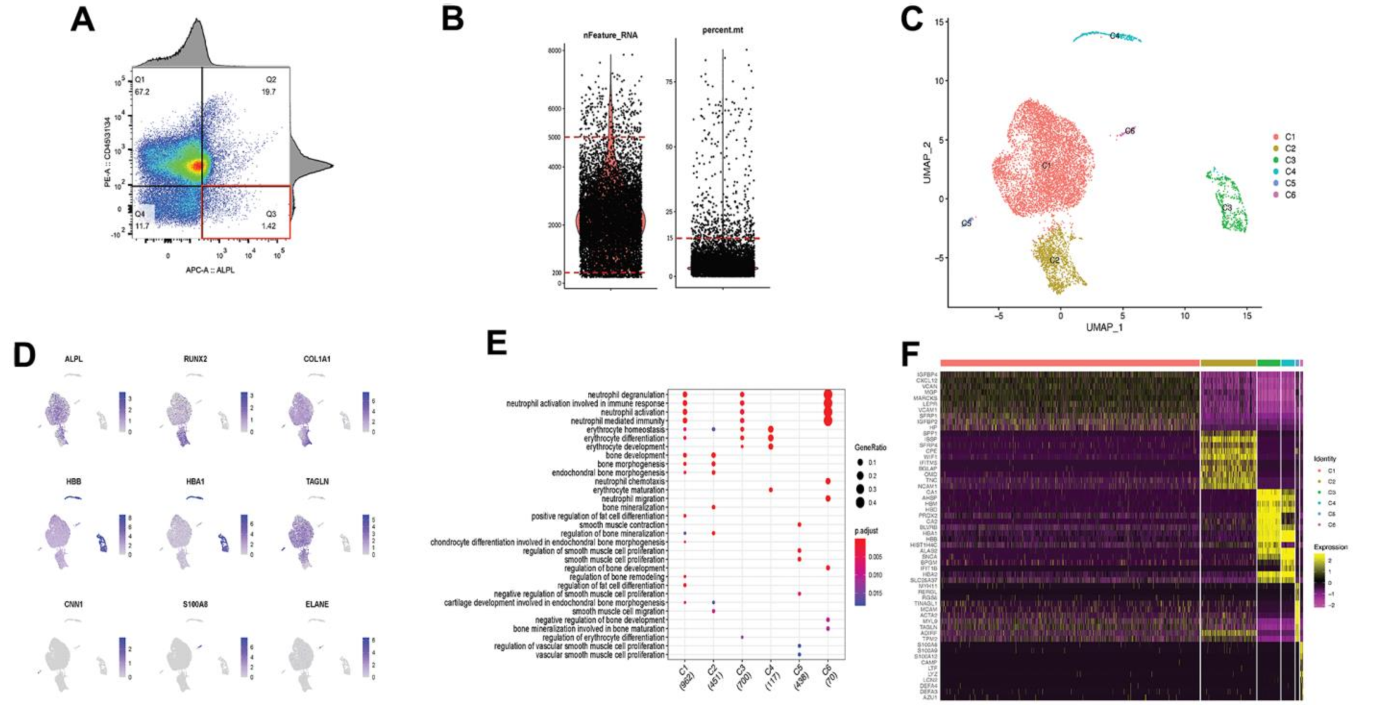

Supplementary Figure 1. Osteoblasts isolation and identification. (**A**) Representative flow cytometry of osteoblasts isolation. The x-axis represents the expression level of ALPL and the y-axis is the expression level of CD45/31/34. Dots represent the cells. Cells in Q3 panel (downer-right) are isolated for downstream analysis. (**B**) Number of genes per cell and the percentage of the transcripts attributed to mitochondrial genes per cell. Dash line represents the thresholds for quality control. (**C**) UMAP dimension reduction of isolated cells, colored by different clusters. (**D**) Expression of known cell markers of each isolated subpopulations embedded on UMAP dimension reduction map. The first three markers (ALPL, RUNX2 and COL1A1) in the first row are the osteogenesis markers. HBB and HBA1 are markers of two nucleated erythrocyte clusters C2 and C3. The last four genes are markers for cluster C4 and C5, respectively. (**E**) GO enrichment for all clusters. The size of dot indicates the gene ratio. The color indicates the adjusted p-value for enrichment analysis. (**F**) Heatmap of gene expression profile of isolated cells, based on the relative gene expression level of top 10 most-significant markers for each cluster. X-axis represents different osteoblast clusters, while y-axis indicates the 10 most-significant markers in each cell cluster.
